# Chitosan Oligosaccharides Alleviate H_2_O_2_-stimulated Granulosa Cell Damage via HIF-1*α* Signaling Pathway

**DOI:** 10.1155/2022/4247042

**Published:** 2022-04-01

**Authors:** Ziwei Yang, Wenli Hong, K. Zheng, Jingyuan Feng, Chuan Hu, Jun Tan, Zhisheng Zhong, Yuehui Zheng

**Affiliations:** ^1^Jiangxi Medical College, Nanchang University, Nanchang 330006, China; ^2^Jiangxi Provincial Key Laboratory of Reproductive Physiology and Pathology, Nanchang 330031, China; ^3^Health Science Center, Shenzhen University, Shenzhen 518000, China; ^4^Medical College, Shantou University, Shantou 515041, China; ^5^Reproductive Medicine Center, Jiangxi Maternal and Child Health Hospital, Nanchang 330006, China; ^6^Department of Reproductive Health, Shenzhen Traditional Chinese Medicine Hospital, Shenzhen 518033, China

## Abstract

Oocyte maturation disorder and decreased quality are the main causes of infertility in women, and granulosa cells (GCs) provide the only microenvironment for oocyte maturation through autocrine and paracrine signaling by steroid hormones and growth factors. However, chronic inflammation and oxidative stress caused by ovarian hypoxia are the largest contributors to ovarian aging and GC dysfunction. Therefore, the amelioration of chronic inflammation and oxidative stress is expected to be a pivotal method to improve GC function and oocyte quality. In this study, we detected the protective effect of chitosan oligosaccharides (COS), on hydrogen peroxide- (H_2_O_2_-) stimulated oxidative damage in a human ovarian granulosa cell line (KGN). COS significantly increased cell viability, mitochondrial function, and the cellular glutathione (GSH) content and reduced apoptosis, reactive oxygen species (ROS) content, and the levels of 8-hydroxy-2′-deoxyguanosine (8-OHdG), 4-hydroxynonenal (4-HNE), hypoxia-inducible factor-1*α* (HIF-1*α*), and vascular endothelial-derived growth factor (VEGF) in H_2_O_2_-stimulated KGN cells. COS treatment significantly increased levels of the TGF-*β*1 and IL-10 proteins and decreased levels of the IL-6 protein. Compared with H_2_O_2_-stimulated KGN cells, COS significantly increased the levels of E_2_ and P_4_ and decreased SA-*β*-gal protein expression. Furthermore, COS caused significant inactivation of the HIF-1*α*-VEGF pathway in H_2_O_2_-stimulated KGN cells. Moreover, inhibition of this pathway enhanced the inhibitory effects of COS on H_2_O_2_-stimulated oxidative injury and apoptosis in GCs. Thus, COS protected GCs from H_2_O_2_-stimulated oxidative damage and apoptosis by inactivating the HIF-1*α*-VEGF signaling pathway. In the future, COS might represent a therapeutic approach for ameliorating disrupted follicle development.

## 1. Introduction

Due to environmental pollution, life pressure, radiation and chemotherapy administered to patients with cancer, and other factors that damage female ovarian function and shorten human life, infertility, premature ovarian failure (POF), and aging have become major medical problems and social problems threatening human health. The speed of ovarian aging is significantly faster than that of other organs of the body. Some scholars have considered ovarian aging as pacemakers of female body aging. Ovarian aging is the initial factor triggering cardiovascular and cerebrovascular diseases, neurodegenerative diseases, and other chronic diseases [1]. Unfortunately, few methods are available to protect the ovary and delay ovarian aging in healthy aged women. In pathological conditions, treatments include administration of gonadotrophin-releasing hormone (GnRH) analogs, transposition of the ovaries outside of the fields of radiation in cancer patients, and cryopreservation of the ovarian cortical tissue. All these methods are either less effective or are still being investigated [2]. The fundamental reason is the mechanism of ovarian aging. Chemicals that potentially protect the oocyte and its feeder cells from injury would be highly useful.

Ovogenesis has a close relationship with the follicle growth and its maturity. Primary oocytes are originated from primordial germ cells [3, 4]. Primary oocytes interact with the surrounding monolayer of follicular cells and gradually developed into primitive follicles. The primitive follicle terminates the dormancy in certain condition; next, it enters the growth state [5]. During the normal follicle development process, oocytes gradually develop and mature, while granulosa cells (GCs) surrounding oocytes also proliferate and differentiate continuously [4]. Nevertheless, more than 99% of mammalian follicles evolve into atresia state in physiological phenomenon, they degenerate in a period of growth and development, and only a few follicles have the ability to accomplish the development process [6–8]. Follicular atresia is attributed to the programmed cell death of ovarian GCs. GCs communicate with and form a protective barrier between the oocyte and follicle microenvironment by transmitting nutrients, energy, and signals to oocytes and play roles in the androgen to estrogen conversion and progesterone synthesis; thus, a decrease in GC quantity and function leads to decreased production of estradiol (E_2_) and progesterone (P_4_) and may trigger follicular atresia [9, 10]. The morphology and quantity of GCs have been used as biomarkers of developmental ability and pregnancy outcomes [11]. Therefore, the disorder of GCs caused by various factors is the key cause of disrupted oocyte maturation.

The mechanisms underlying decreased GC function and subsequent ovarian aging are not wholly clear. Previous studies have shown that chronic inflammation and oxidative stress (OS) caused by ovarian hypoxia are the main causes of ovarian aging and GC dysfunction [12, 13]. Hypoxia, via a hypoxia-inducible factor-1*α*- (HIF-1*α*-) dependent manner, promotes GC expression of more than 70 downstream genes (including nuclear factor kappa-B (NF-*κ*B), nitric oxide synthase (NOS), and VEGF), resulting in the overproduction of IL-6 and ROS [14–18]. ROS promote the formation of DNA adducts such as 8-hydroxy-2′-deoxyguanosine (8-OHdG) and lipid peroxidation such as 4-hydroxynonenal (4-HNE). Both of these adducts and inflammatory factors jointly cause the accumulation of DNA damage, epigenetic changes, abnormal gene expression and altered cell signaling pathways in GCs, leading to cell and organ aging [19–22]. At present, our group and others' studies have shown that ovarian aging is delayed by improving mild inflammatory and OS environment in the ovary [23, 24]. Therefore, the search for natural materials and chemicals to improve the inflammatory and OS environment of GCs is expected to provide a novel method to rejuvenate the ovary via GC function restoration. COS might be the target chemical meeting these properties. COS is a derivative of chitin that is mainly derived from crustaceans, fungi, insects, and algae cell membranes [25]. COS, water-soluble alkaline polysaccharide with positively charged, has good biocompatibility and is widely used in food, medicine, the chemical industry, environmental protection, cosmetics, agriculture, and other applications [26]. It is known as the “sixth life element” after protein, fat, sugar, vitamin, and mineral in the biomedical field. COS can be used as a functional food and is known as “soft gold.” COS is an oligomer composed of *β*-(1 ➔ 4)-linked d-glucosamine, and it is an oligosaccharide obtained by the hydrolysis of chitosan (CTS) [27]. COS has distinct characteristics, such as a low molecular weight, noncytotoxicity, good water solubility, and easy absorption in the intestine. Meanwhile, COS possesses various diverse biological features, including anti-inflammatory, radical-scavenging, antioxidant, and antidiabetic effects [28]. Currently, products of health care and skincare and other applications have been applied COS as a supplement.

The role of reactive oxygen species (ROS) concentrations in follicular fluid in gynecological diseases and assisted reproduction is relevant to its role in reproductive outcomes [29, 30]. Patients with premature ovarian failure, polycystic ovarian syndrome (PCOS), and physiologically aging ovaries experience a decrease in follicle quality and disrupted oocyte maturation. These tissues are all in chronic low-grade inflammation state caused by OS [31–33]. Tiwari and Chaube found that a gentle increase in ROS levels in the follicle is advantageous for meiotic resumption and first polar body extrusion [34]. In contrast, the excess accumulation of ROS such as H_2_O_2_ and superoxide anion radicals leads to OS, and progressive OS generally induces an inflammatory state [35, 36]. High levels of ROS in the follicle microenvironment prevent the recovery of meiosis of diploid terminated oocyte and GC function, causing poor communication between GCs and oocytes by reducing the supply of nutrition, and eventually negatively affect oocyte quality and maturation [37–40].

Hypoxia-inducible factor (HIF) expression is induced by hypoxia and by other pathological environments associated with inflammation, aging, infectious microorganisms, and tumors [41–44]. It is composed of three subunits: 1*α*, 2*α*, and 1*β*. HIF-1*α* is the main subunit that is widely expressed and regulates a variety of target genes, such as glucose transporters, and VEGF [45]. Recent research has reported that inflammation may lead to cumulate HIF-1*α* protein in macrophages via a mechanism relative to ROS [46]. In addition, KC7F2 is an HIF-1*α* protein inhibitor but does not affect the protein degradation rate or mRNA transcription. TGF-*β* (transforming growth factor-*β*), along with its superfamily number anti-Müllerian hormone (AMH), bone morphogenetic protein-15 (BMP-15), and growth differentiation factor-9 (GDF-9), plays important roles in influencing the developing follicle microenvironment through autocrine and paracrine manner [47]. In ovary and other organs, hypoxia significantly inhibited GSH activities and superoxide dismutase (SOD) and enhanced the production of malondialdehyde (MDA) [48–50].

However, it's not clear whether COS has a role in GC development and function by controlling chronic low-grade inflammation and OS. We studied the direct effects of different COS concentrations on a human ovarian granulosa cell line (KGN) as a first step to examine the potential protective effect of COS on H_2_O_2_-stimulated GC dysfunction. Also, we studied the effects of COS on KGN cells mediated by the HIF-1*α* signaling pathway.

## 2. Materials and Methods

### 2.1. Experimental Set-Up

The human ovarian granulosa cell line (KGN) was obtained from Dr. Yang Zou (Jiangxi Maternal and Child Health Hospital, China). The culture medium used was DMEM/F12 (Wisent, China) with 10% FBS (Gibco, USA) and 1% antibiotics (Solarbio, China). KGN cells were incubated with 5% CO_2_ at 37°C.

*Phase A*: we investigated the H_2_O_2_-stimulted KGN OS model. KGN cells were seeded in 96-well plates at a density of 8 × 10^3^ cells/well. Then, cells were treated with 100, 200, 400, or 800 *μ*M H_2_O_2_ (Wako Pure Chemical Industries, China) for 1, 2, 3, or 4 h. The OD value was detected using the CCK-8 method, and the cell survival (%) was calculated. A cell survival close to 50% was required to determine the optimal concentration and time for subsequent experiments.

*Phase B*: we investigated the dose- and time-dependent effects of a broad range of concentrations of COS (0, 100, 200, and 300 *μ*g/ml) (Glycobio, China, purity ≥90%) on H_2_O_2_-stimulated OS damage in KGN cells.

*Phase C*: the optimal concentration of COS was studied to explore the mechanism underlying the protective effect.

### 2.2. Enzyme-Linked Immunosorbent Assay (ELISA)

The centrifuged supernatant of the medium was determined using ELISA kits (Westang, China) for the levels of IL-6, IL-10 (cell lysates), E_2_, and P_4_, according to the manufacturer's recommendations.

### 2.3. Immunofluorescence Staining

#### 2.3.1. Measurement of the Mitochondrial Quantity

KGN cells were incubated with 200 nM MitoTracker (AAT Bioquest, USA) diluted with medium (without FBS) for 30 min at 37°C to specifically assess mitochondrial numbers.

#### 2.3.2. Detection of ROS Production

The Reactive Oxygen Species Assay Kit (Solarbio, China) was used to determine ROS level. After COS treatment, KGN cells were loaded with 10 *μ*M DCFH-DA in FBS-free medium for 20 min at 37°C. Then, the medium containing DCFH-DA was removed, and KGN cells were washed to remove unconjugated DCFH-DA.

#### 2.3.3. Measurement of the Mitochondrial Membrane Potential (MMP, *ΔΨ*m)

MMP was detected by MMP assay kit with JC-1 (Beyotime, China). KGN cells were incubated with 1 ml staining working solution (8 ml ultrapure water + 50 *μ*l JC − 1 (200X) + 2 ml JC − 1 staining buffer (5X)) and 1 ml FBS-free medium per well (6-well plates) at 37°C in the dark for 20 min, then washed with JC-1 iced staining buffer (1X), and maintained in FBS-free medium while being examined.

All of the fluorescence intensity was examined under an Olympus 1X71 microscope and analyzed with ImageJ. The results are from 3 replications.

### 2.4. Cell Counting Kit-8

Cell viability was measured using the Cell Counting Kit-8 assay (CCK-8, APExBIO, USA) in 96-well plates at a density of 8 × 10^3^ cells/well. The mixture liquid (10 *μ*l CCK − 8 assay reagent + 90 *μ*l FBS − free medium) was added to each well and incubated in the dark for 2 h at 37°C. The optical density (OD) was assessed by a microplate reader (Bio-Rad, USA).

### 2.5. Flow Cytometry

KGN cells were resuspended in annexin V binding buffer with FITC-conjugated annexin V (Bestbio, China). Then, 10 *μ*l of propidium iodide was added. A flow cytometer was used to analyze the KGN cells (Beckman Coulter, USA).

### 2.6. Western Blotting

KGN cells were incubated with lysis buffer supplemented with a protease inhibitor cocktail (Solarbio, China) on ice. The supernatant was collected after centrifugation at 4°C for 15 min. Protein concentrations were measured using the DC protein assay (Bio-Rad Laboratories Inc.). Equal amounts of total protein (25 *μ*g) were separated on 10% SDS–PAGE gels (Boster, USA) and transferred onto PVDF membranes (Immobilon-P, USA). Membranes were blocked with 5% nonfat milk in TBST and incubated with primary antibodies against TGF-*β*1 (1 : 500; ab92486; Abcam), HIF-1*α* (1 : 500; 66730; Proteintech), VEGF*α* (1 : 1,000; 66828-1; Proteintech), 4-HNE (1 : 1,000; ab46545; Abcam), 8-OHdG (1 : 1,000; 251640; Abbiotec LLC), or *β*-actin (1 : 1,000; AC006; ABclonal) overnight at 4°C. The corresponding secondary antibody (1 : 3,000; RS0001; ImmunoWay) was incubated with the membrane at room temperature for 1 h. Immunoreactive bands were detected using a western blotting luminescence reagent (ECL) kit (Tiangen, China) and photographed with ChemiDoc XRS (Bio-Rad, USA). The protein band was analyzed with ImageJ software.

### 2.7. RNA Extraction and RT–qPCR

KGN cell RNA was extracted with TRIzol® reagent (Tiangen, China). cDNAs were generated by reverse transcription with a PrimeScript RT Reagent Kit (Takara, Japan). RT-qPCR using an ABI 7500 real-time PCR instrument (Biosystems, USA) with SYBR® Green PCR Master Mix (Takara, Japan) was performed. The primers for the reference genes were HIF-1*α* (forward, 5′-AACATAAAGTCTGCAACATGGAAG-3′ and reverse, 5′-TTTGATGGGTGAGGAATGGG-3′), VEGF (forward, 5′-AGTCCAACATCACCATGCAG-3′ and reverse, 5′-TTCCCTTTCCTCGAACTGATTT-3), and GAPDH (forward, 5′-ACATCGCTCAGACACCATG-3′ and reverse, 5′-TGTAGTTGAGGTCAATGAAGGG-3′). The specificity was validated by performing a melting curve analysis, and experiments were repeated at least three times.

### 2.8. SA-*β*-Gal Assay

The SA-*β*-gal assay was carried out according to the manufacturer's instructions (Beyotime, China). Freshly collected KGN cells were fixed for 15 min with *β*-galactosidase fixative at room temperature, washed, and stained with X-gal solution overnight at 37°C. Cells were photographed under an Olympus 1X71 microscope. The percentage of blue SA-*β*-gal positive cells was determined using ImageJ software.

### 2.9. GSH

The cellular total GSH were measured using a GSH Assay Kit (Beyotime, China). KGN cells were collected after centrifugation. Then, the supernatant was removed. M solution for protein removal was added, and the volume was three times the cell pellet volume. The samples were rapidly freeze-thawed three times. Afterward, suspension was centrifuged. The supernatant was collected to measure the total GSH content.

### 2.10. Statistical Analysis

All data (three independent cultures) are presented as the means ± SEM (standard errors of the means). Statistical analyses were performed using Student's *t*-test and one-way ANOVA with SPSS software. *P* < 0.05 were considered statistically significant.

## 3. Results

### 3.1. H_2_O_2_ Inhibited KGN Cell Survival in a Concentration- and Time-Dependent Manner

**Figure 1:**
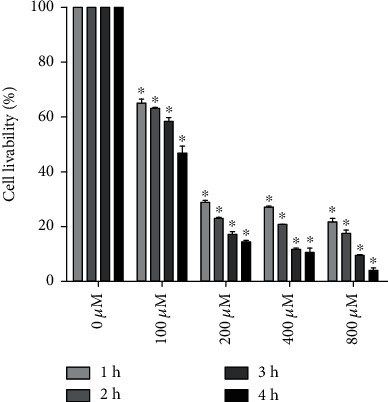
H_2_O_2_ inhibited KGN cell survival in a concentration- and time-dependent manner. Data are presented as the means ± SEM (*n* = 3). ^∗^*P* < 0.05 vs. the 0 *μ*M group.

CCK-8 results showed that H_2_O_2_ inhibited the cell survival of KGN cells in vitro. Various H_2_O_2_ concentrations (100, 200, 300, and 400 *μ*M) showed concentration- and time-dependent inhibition of cell survival (Figure 1). When cells were treated with 100 *μ*M H_2_O_2_ for 4 h, the cell survival rate was 53.25 ± 3.76%, which was closest to the IC50. Therefore, 100 *μ*M and 4 h were selected for subsequent experiments as the optimal concentration and time, respectively.

### 3.2. COS Increased Viability and Inhibited Apoptosis in KGN Cells under Oxidative Stress

**Figure 2:**
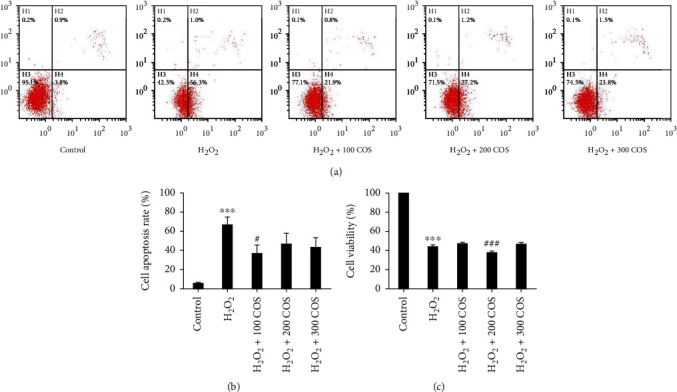
Effect of COS treatment on cell viability and apoptosis in H_2_O_2_-stimulated KGN cells. (a) Cell apoptosis rate. (b) Quantification of cell apoptosis rate. (c) Cell viability. Data are presented as the means ± SEM (*n* = 3). ^∗^*P* < 0.05, ^∗∗^*P* < 0.01, or ^∗∗∗^*P* < 0.001 vs. the control group; ^#^*P* < 0.05, ^##^*P* < 0.01, or ^###^*P* < 0.001 vs. the H_2_O_2_ group.

H_2_O_2_ obviously reduced cell viability (Figure 2(c), *P* < 0.001), but the viability of COS treatment groups was higher than that of the H_2_O_2_ group, except the 200 COS group, but the differences were not significant (*P* > 0.05). In Figure 2(a), annexin V and PI assays were conducted to evaluate apoptosis. The percentage of apoptotic cells was calculated from H2 (late stage of apoptosis) and H4 (early stage of apoptosis). In Figure 2(b), H_2_O_2_ increased the proportion of apoptotic cells (*P* < 0.001). After treatment with different concentrations of COS, the percentage of apoptotic cells was significantly decreased (*P* < 0.05 in the H_2_O_2_+100 COS group). These results support that COS protects KGN cells from apoptosis stimulated by H_2_O_2_.

### 3.3. COS Increased Levels of the TGF-*β*1 and IL-10 Proteins and Steroidogenesis and Decreased Levels of the IL-6 Protein in H_2_O_2_-Stimulated KGN Cells

**Figure 3:**
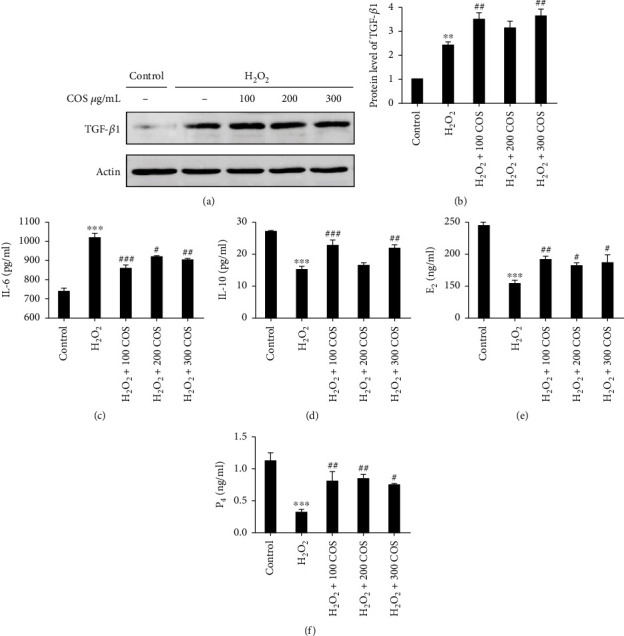
The expression of TGF-*β*1, IL-6, IL-10, E_2_, and P_4_ after COS treatment in H_2_O_2_-stimulated KGN cells. (a) The protein expression of TGF-*β*1. (b) The statistics histogram of western blotting was expressed as band density normalized versus ACTIN. (c–f) The levels of IL-6, IL-10, E_2_, and P_4_. Data are presented as the means ± SEM (*n* = 3). ^∗^*P* < 0.05, ^∗∗^*P* < 0.01, or ^∗∗∗^*P* < 0.001 vs. the control group; ^#^*P* < 0.05, ^##^*P* < 0.01 or ^###^*P* < 0.001 vs. the H_2_O_2_ group.

As depicted in Figure 3(a), H_2_O_2_ increased TGF-*β*1 levels (*P* < 0.01), similar to previous studies [51, 52]. The increase in IL-6 levels and decrease in IL-10 expression induced by H_2_O_2_ (Figures 3(c) and 3 (d), *P* < 0.001, respectively) were substantially reversed by COS. Additionally, COS significantly increased TGF-*β*1 protein expression (*P* < 0.01 in the H_2_O_2_+100 COS/300 COS group).

H_2_O_2_ decreased steroidogenesis in KGN cells (Figures 3(e) and 3(f), *P* < 0.001, respectively). The levels of secreted E_2_ and P_4_ were increased by COS treatment at different doses ranging from 100 to 300 *μ*g/ml.

Based on these results, COS treatment exerts a positive effect on steroid production and induces anti-inflammatory and proliferative activities that help KGN cells recover from OS.

### 3.4. COS Inhibited Oxidative Stress in H_2_O_2_-Stimulated KGN Cells

**Figure 4:**
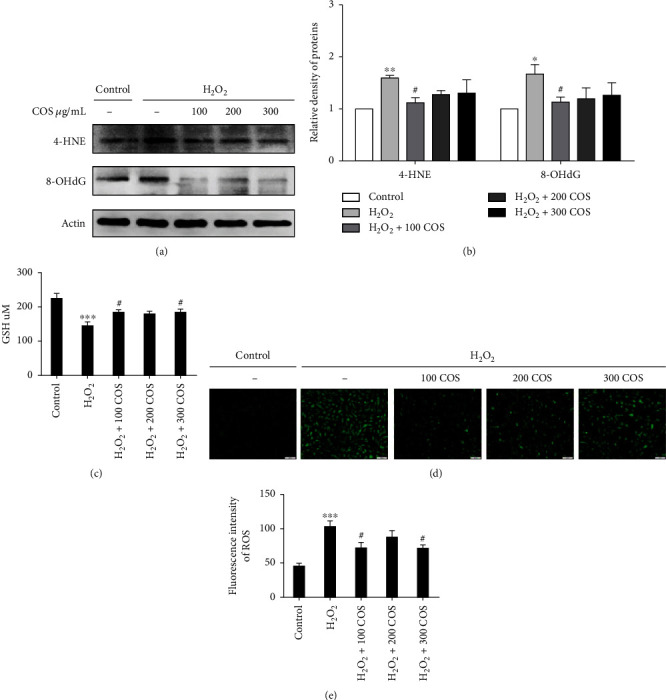
COS inhibited oxidative stress in H_2_O_2_-stimulated KGN cells. (a) The protein expressions of 4-HNE and 8-OHdG after COS treatment. (b) The statistics histogram of western blotting is expressed as band density normalized versus actin. (c) Influence of COS on cellular GSH. (d) The cellular production of ROS determined by a fluorescent probe. (e) The statistics histogram of immunofluorescence expressed fluorescent intensity. Data are presented as the means ± SEM (*n* = 3). ^∗^*P* < 0.05, ^∗∗^*P* < 0.01, or ^∗∗∗^*P* < 0.001 vs. the control group; ^#^*P* < 0.05, ^##^*P* < 0.01, or ^###^*P* < 0.001 vs. the H_2_O_2_ group. Scale bar: 100 *μ*m.

The levels of 4-HNE, 8-OHdG, ROS, and GSH were evaluated as markers of OS. In Figures 4(a) and 4(d), the levels of 4-HNE and 8-OHdG and ROS release were markedly increased in KGN cells exposed to H_2_O_2_ (*P* < 0.01 for 4-HNE, *P* < 0.05 for 8-OHdG, and *P* < 0.001 for ROS). The increased production of 4-HNE, 8-OHdG, and ROS was significantly attenuated in the H_2_O_2_+100 COS group (*P* < 0.05 for 4-HNE/8-OHdG, *P* < 0.05 for ROS). The results presented in Figure 4(c) proved that the H_2_O_2_-stimulated decrease in GSH activity (*P* < 0.001) was mitigated by the COS treatment (*P* < 0.05 in the H_2_O_2_+100 COS/300 COS group). Overall, these data show that COS restored the function of H_2_O_2_-stimulated KGN cells through attenuating OS level.

### 3.5. COS Improved Mitochondrial Function in H_2_O_2_-Stimulated KGN Cells

**Figure 5:**
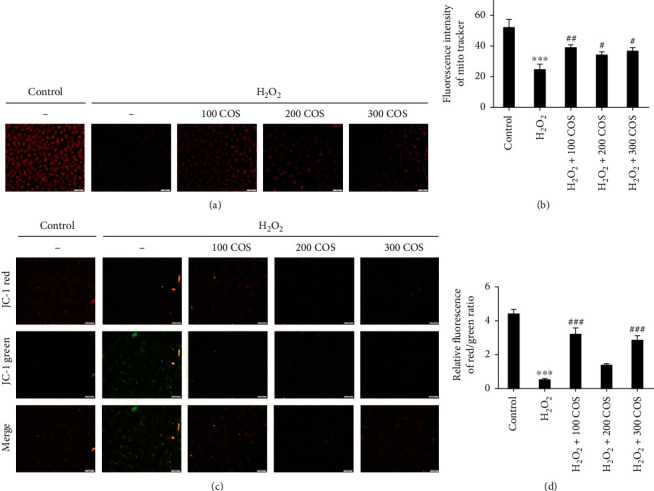
COS improved mitochondrial function in H_2_O_2_-stimulated KGN cells. (a) MitoTracker was stained on KGN by immunofluorescence. (b) The statistics histogram of immunofluorescence expressed fluorescent intensity. (c) JC-1 red, JC-1 green, and merge images. (d) Statistics data were expressed in terms of the ratio of JC-1 red to JC-1 green. Data are presented as the means ± SEM (*n* = 3). ^∗^*P* < 0.05, ^∗∗^*P* < 0.01, or ^∗∗∗^*P* < 0.001 vs. the control group; ^#^*P* < 0.05, ^##^*P* < 0.01, or ^###^*P* < 0.001 vs. the H_2_O_2_ group. Scale bar: (a): 100 *μ*m; (c): 50 *μ*m.

Mitochondria are one of the most important organelles and indicators of cell function. The mitochondrial numbers were determined by coloading the cells with a mitochondrion-specific probe, MitoTracker. H_2_O_2_ reduced MitoTracker staining. (Figure 5(a), *P* < 0.001). After COS treatment, the fluorescence intensity was noticeably increased (*P* < 0.01 in the H_2_O_2_+100 COS group; *P* < 0.05 in the H_2_O_2_+200/300 COS group). Thus, COS increased mitochondrial numbers. We also assessed MMP using JC-1 and measuring the red/green ratio. A decrease of MMP in KGN cell exposure to H_2_O_2_ was observed (Figure 5(c), *P* < 0.001). Treatment with COS rescued the cells from the loss of MMP (*P* < 0.001 in the H_2_O_2_+100 COS/300 COS group). These data indicate that COS can improve mitochondrial quantity and MMP in H_2_O_2_-stimulated KGN cells.

### 3.6. COS Inactivated the HIF-1*α*/VEGF Pathway, and HIF-1*α* Inhibition Reversed the Protective Effects of COS on H_2_O_2_-Stimulated KGN Cells

**Figure 6:**
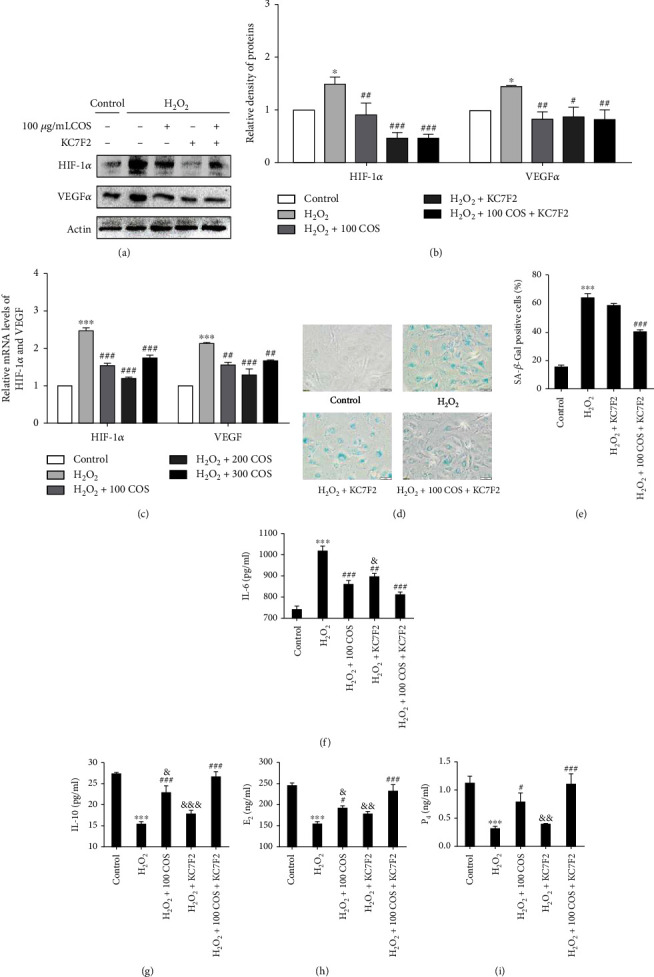
The effect of the COS treatment on the HIF-1*α*/VEGF pathway in H_2_O_2_-stimulated KGN cells. (a) The protein expressions of HIF-1*α* and VEGF. (b) The statistics histogram of western blotting is expressed as band density normalized versus actin. (c) The mRNA expressions of HIF-1*α* and VEGF. (d) Determination of senescence in KGN cells by staining for SA-*β*-gal (200X). (e) Statistical analysis of quantification of the SA-*β*-gal-positive cells. (f–i) The levels of IL-6, IL-10, E_2_, and P_4_. Data are presented as the means ± SEM (*n* = 3). ^∗^*P* < 0.05, ^∗∗^*P* < 0.01, or ^∗∗∗^*P* < 0.001 vs. the control group; ^#^*P* < 0.05, ^##^*P* < 0.01, or ^###^*P* < 0.001 vs. the H_2_O_2_ group; ^&^*P* < 0.05, ^&&^*P* < 0.01, or ^&&&^*P* < 0.001 vs. the H_2_O_2_+100 COS+KC7F2 group.

**Figure 7:**
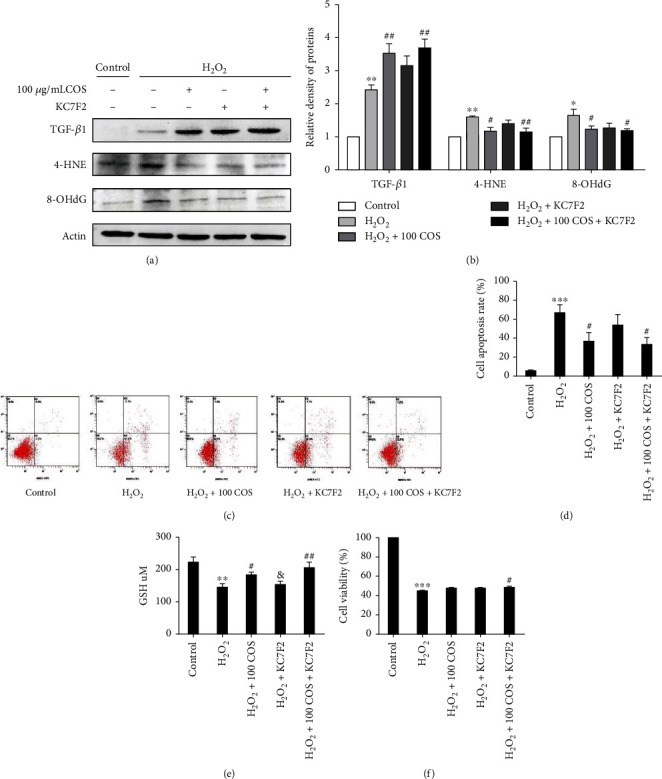
The effect of the COS treatment on the HIF-1*α*/VEGF pathway in H_2_O_2_-stimulated KGN cells. (a) The levels of TGF-*β*1, 4-HNE, and 8-OHdG. (b) The statistics histogram of western blotting are expressed as band density normalized versus actin. (c) Cell apoptosis rates. (d) Quantitative results of KGN cell apoptosis rate. (e, f) Cellular GSH content and cell viability. Data are presented as the means ± SEM (*n* = 3). ^∗^*P* < 0.05, ^∗∗^*P* < 0.01, or ^∗∗∗^*P* < 0.001 vs. the control group; ^#^*P* < 0.05, ^##^*P* < 0.01, or ^###^*P* < 0.001 vs. the H_2_O_2_ group; ^&^*P* < 0.05, ^&&^*P* < 0.01, or ^&&&^*P* < 0.001 vs. the H_2_O_2_+100 COS+KC7F2 group.

**Figure 8:**
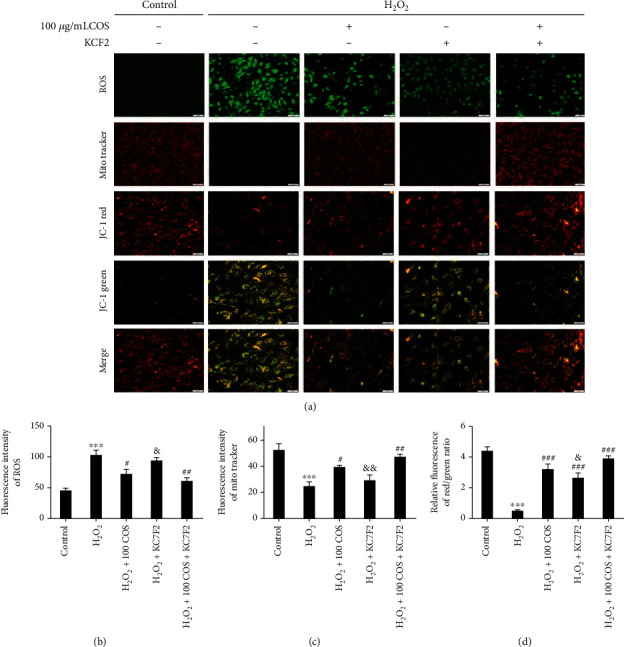
The effect of the COS treatment on the HIF-1*α*/VEGF pathway in H_2_O_2_-stimulated KGN cells. (a) Production of ROS determined by a fluorescent probe. (b) MitoTracker was stained on KGN cells by immunofluorescence. (c) JC-1 red, JC-1 green, and merge image. (d, e) The statistics histogram of immunofluorescence expressed fluorescent intensity. (f) Statistics data were expressed in terms of the ratio of JC-1 red to JC-1 green. Data are presented as the means ± SEM (*n* = 3). ^∗^*P* < 0.05, ^∗∗^*P* < 0.01, or ^∗∗∗^*P* < 0.001 vs. the control group; ^#^*P* < 0.05, ^##^*P* < 0.01, or ^###^*P* < 0.001 vs. the H_2_O_2_ group; ^&^*P* < 0.05, ^&&^*P* < 0.01, or ^&&&^*P* < 0.001 vs. the H_2_O_2_+100 COS+KC7F2 group. Scale bar: (a) ROS/MitoTracker: 100 *μ*m; JC-1 red/JC-1 green/merge: 50 *μ*m.

Western blotting analysis revealed that H_2_O_2_ exposure markedly increased levels of the HIF-1*α* and VEGF*α* proteins in KGN cells (Figure 6(a), *P* < 0.05, respectively), and COS inhibited the increases in HIF-1*α* and VEGF*α* protein levels induced by H_2_O_2_ in KGN cells. When HIF-1*α* was inhibited by KC7F2, it also reversed the increases in VEGF*α*, IL-6 (Figure 6(f)), and TGF-*β*1 protein levels (Figure 7(a)) and decreases in the levels of IL-10 (Figure 6(g)), E_2_ (Figure 6(h)), P_4_ (Figure 6(i)), MitoTracker staining (Figure 8(a)), GSH content (Figure 7(e)), and cell viability (Figure 7(f)) induced by H_2_O_2_, as well as the increase in the level of senescence-associated *β*-galactosidase (SA-*β*-gal) (Figure 6(d)). Furthermore, the increased levels of 4-HNE and 8-OHdG (Figure 7(a)), ROS release (Figure 8(a)), and cell apoptosis rate (Figure 7(c)) induced by H_2_O_2_ were all prevented by KC7F2.

COS and KC7F2 exerted synergistic effects on all of these outcomes. Based on these results, the HIF-1*α*-VEGF signaling pathway mediated the protective effects of COS on H_2_O_2_-stimulated KGN cells.

## 4. Discussion

Aging, mental stress, endocrine disorders, and chemo- and/or radiotherapy all exert substantial effects on female fertility [53–55]. Currently, few methods are available to preserve ovarian function in human being. Furthermore, these methods are less effective and still under investigated in experimental research. Chemicals that potentially protect the follicle of oocyte and GCs from injury might be the next female fertility-reserving medicine. A vital antioxidant and a forceful free radical scavenger, COS might be this type of agent.

**Figure 9:**
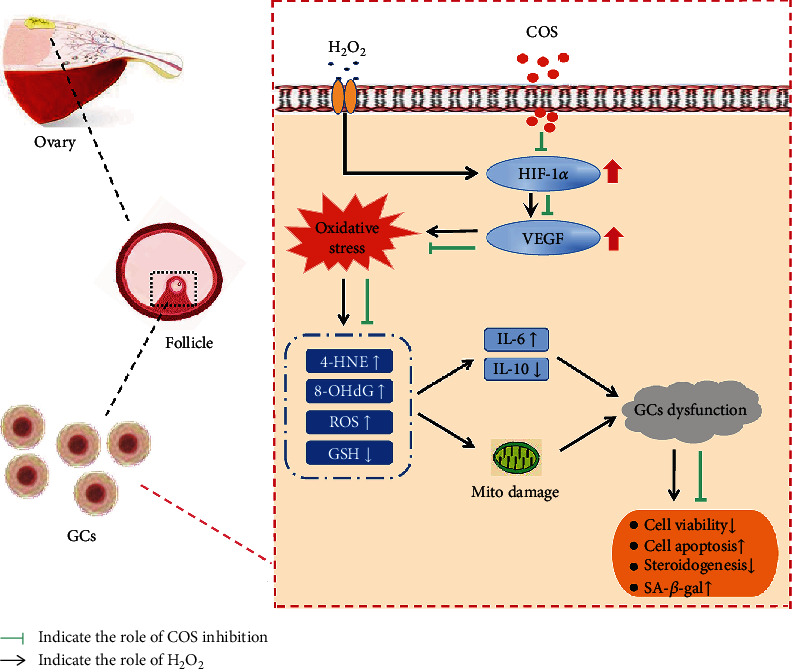
COS attenuated oxidative and inflammatory injury in H_2_O_2_-stimulated KGN cells. The protective effects of COS were manifested in inactivating the HIF-1*α*-VEGF signaling pathway, attenuating OS, decreasing the production of inflammatory cytokines, and improving mitochondrial damage caused by H_2_O_2_ in KGN cells.

GCs promote oocyte maturation and protect oocytes from OS damage via supplying essential nutrients and maturation-promoting factors; they play crucial roles in folliculogenesis [56]. GCs are susceptible to ROS, which are byproducts produced in the course of the citric acid cycle. The generation of endogenous ROS is through the following: one is various metabolic processes, and the other is the electron transport chain. Mitochondria are intimately involved in cellular respiration and metabolism, and thus, they are the main generator of endogenous ROS and the main target organelle for OS damage. There are two main methods to remove ROS. One is the internal antioxidant system, which consists of enzyme such as SOD and the nonenzymatic antioxidant such as GSH [49, 50]. GSH is a major cellular antioxidant, also recognized key indicator to determine the degree of OS [50]. The other is exogenous antioxidant from supplement to improve the antioxidant capacity [57]. Obviously, it leads to a better effect that endogenous and exogenous antioxidant methods simultaneously remove ROS to resist oxidant damage. ROS is not only considered to be the most critical induction factor of cell damage caused by oxidative stress; meanwhile, free radicals mainly attack macromolecules such as biofilms, membrane system, DNA, and protein. Free radicals easily react with unsaturated fatty acids on the membrane then produce lipid radicals, destroying the original membrane structure [58]. The levels of ROS are markedly accumulative after stimulation and induction OS in GCs, leading to GC dysfunction [59]. Hence, ROS-induced injury of OS and GC decline in quantity are deemed the primary etiological factors of ovarian dysfunction. In this study, H_2_O_2_ was applied to induce OS and GC apoptosis [60–62]. We found that COS-mediated GC protection depends on improved mitochondrial function and a decrease in apoptosis. Therefore, our results suggest that COS inhibits OS-induced damage in GCs by ameliorating mitochondrial damage. Numerous animal studies have shown that OS and chronic inflammation promote each other in a vicious cycle: OS leads to an increase in ROS production through NOD-like receptor 3 (NLRP3) and NF-*κ*B, resulting in a series of inflammatory responses and increased secretion of IL-1*β*, IL-8, and TNF-*α*, which in turn promote OS and accelerate the organ aging process [63]. So we evaluated the protective effect of COS on H_2_O_2_-oxidative damage and cytokine production in KGN cells. COS significantly increased cell viability, the cellular GSH content, and mitochondrial function and reduced ROS production; the levels of 8-OHdG, 4-HNE, IL-6, HIF-1*α*, and VEGF; and cell apoptosis. COS also significantly increased TGF-*β*1 and IL-10 expression in the H_2_O_2_-stimulated KGN cells, significantly increased E_2_ and P_4_ levels, and decreased in SA-*β*-gal protein expression, implying that COS attenuated oxidative and inflammatory injury in H_2_O_2_*-*stimulated KGN cells (Figure 9). Partial results showed that the effect of the H_2_O_2_+200 COS group was lower than that of the other two groups, but the *P* values between the most optimal concentration group (the H_2_O_2_+100 COS group) and the H_2_O_2_+200 COS group were not significant except the results in IL-6 and IL-10. It may be related to the higher sensitivity of ELISA than western blotting in quantifying. Meanwhile, it proved that COS was not concentration-dependent in the KGN OS model.

We were very curious about the triggers of inflammation and OS-induced damage. What is the mechanism of action? We speculated that the mechanism may be related to ovarian hypoxia. Hypoxia, the imbalance between the oxygen supply and demand, is the factor that induces chronic inflammation and OS. Due to the unique tissue construct and large volume of oocyte, the ovary is a hypoxic organ. On the one hand, with the growth and development of follicular oocytes and the proliferation and division of GCs, the oxygen demand gradually increases; on the other hand, continuous ovulation leads to an increase in the amount of fibrous connective tissue and a significant decrease in the number of blood vessels in the ovary, which leads to a decreased oxygen and blood supply in the ovary with aging. In addition, the chronic low-grade inflammatory response caused by repeated ovulation and the accompanying OS further aggravate the imbalance between supply and demand, which reduces the oxygen concentration in the ovary to approximately 1.3%-5.5% [64, 65]. This finding may be an important explanation for the significantly faster ovarian aging rate than that in other organs of the body. Molinari et al.'s transcriptome analysis of human cumulus cells also revealed that hypoxia is a marker and important determinant of follicular senescence [66]. The activity of hydroxylase and the degradation of HIF-1*α* are prevented in hypoxic circumstances. Stimulation with TNF-*α* or ROS (such as H_2_O_2_) leads to enhance NF-*κ*B transcription, which binds to a distinct element at −197/188 bp of the HIF-1*α* promoter, thus synthetizing HIF-1*α* mRNA and protein [67–69]. Hypoxia increases glycolysis metabolism through HIF-1*α*; the HIF-1*α*-dependent pathway activates monocytes/macrophages and TH1/TH17 cells through nuclear transcription factor NF-*κ*B and increases the generation of inflammatory factors such as IL-6, IFN7, and TNF-*α*; ROS can activate NF-*κ*B, which is a significant transcription factor in the complete progress of HIF-1 activation, leading to chronic inflammation of the ovary [49, 70–74]. The relationship between NF-*κ*B and HIF-1 is of major importance for inflammatory diseases. HIF-1 can also activate NF-*κ*B. HIF-1*α* is a direct target gene of NF-*κ*B [75, 76]. Hypoxia induces excess ROS production, which may cause OS-induced damage to the ovary, including the oxidation of proteins, lipids, and DNA. Oxidative damage promotes the production of adducts: 8-OHdG and 4-HNE [77–79]. Both of the adducts, together with inflammatory factors, cause the accumulation of epigenetic alterations, DNA damage, abnormal gene expression, and dysregulation of cell signaling pathways, leading to cell and organ aging [80–83].

Recently, the Guanghui Liu Laboratory at the Chinese Academy of Sciences (CAS), Shang Fu Laboratory at Peking University, and Qu Jing research group at CAS collaborated and used high-precision single-cell transcriptome sequencing technology to draw the first map of cynomolgus monkey ovarian cell aging and used a human ovarian cell research system at the same time. Aging leads to an imbalance in the cell type-specific redox regulatory network in the ovary, and the decrease in the antioxidant capacity associated with aging is one of the main characteristics of aging in the primate ovary [83]. In this study, we found that COS caused significant inactivation of the HIF-1*α*-NF-*κ*B-VEGF pathway in H_2_O_2_-stimulated KGN cell. Furthermore, inhibition of this pathway strengthened the inhibitory effects of COS on H_2_O_2_-stimulated OS damage and apoptosis in KGN cells.

## 5. Conclusion

In this study, by comparing the oxidative stress damage, we concluded that COS exerts protective effects on H_2_O_2_-stimulated oxidative damage and apoptosis in GCs via the inactivation of the HIF-1*α*-NF-*κ*B–VEGF signaling pathway. Therefore, COS might be an agent for ovarian pathology therapy through its regulatory effect on follicular development.

## Data Availability

The data used to support the finding of this study are included within the article.

## Abbreviations

GCs: Granulosa cells
COS: Chitosan oligosaccharides
H_2_O_2_: Hydrogen peroxide
KGN: Granulosa cell line
GSH: Glutathione
ROS: Reactive oxygen species
8-OHdG: 8-Hydroxy-2′-deoxyguanosine
4-HNE: 4-Hydroxynonenal
HIF-1*α*: Hypoxia-inducible factor-1*α*
VEGF: Vascular endothelial-derived growth factor
TGF-*β*1: Transforming growth factor *β*-1
SA-*β*-Gal: Senescence-associated *β*-galactosidase
POF: Premature ovarian failure
GnRH: Gonadotrophin-releasing hormone
OS: Oxidative stress
CTS: Hydrolysis of chitosan
PCOS: Polycystic ovarian syndrome
MMP: Mitochondrial membrane potential
NLRP3: NOD-like receptor 3
NF-*κ*B: Nuclear factor kappa-B
NOS: Nitric oxide synthase
SOD: Superoxide dismutase
MDA: Malondialdehyde
GDF-9: Growth differentiation factor-9
BMP-15: Bone morphogenetic protein-15
AMH: Anti-Müllerian hormone
Nrf2: Nuclear factor erythroid 2-related factor 2.
